# WINCS Harmoni: Closed-loop dynamic neurochemical control of therapeutic interventions

**DOI:** 10.1038/srep46675

**Published:** 2017-04-28

**Authors:** Kendall H. Lee, J. Luis Lujan, James K. Trevathan, Erika K. Ross, John J. Bartoletta, Hyung Ook Park, Seungleal Brian Paek, Evan N. Nicolai, Jannifer H. Lee, Hoon-Ki Min, Christopher J. Kimble, Charles D. Blaha, Kevin E. Bennet

**Affiliations:** 1 Department of Neurologic Surgery, Mayo Clinic, Rochester, MN 55905, United States of America; 2 Department of Physiology and Biomedical Engineering, Mayo Clinic, Rochester, MN 55905, United States of America; 3 Department of Physical Medicine and Rehabilitation, Mayo Clinic, Rochester, MN 55905, United States of America; 4 Mayo Graduate School, Mayo Clinic, Rochester, MN 55905, United States of America; 5 Division of Engineering, Mayo Clinic, Rochester, MN 55905, United States of America

## Abstract

There has been significant progress in understanding the role of neurotransmitters in normal and pathologic brain function. However, preclinical trials aimed at improving therapeutic interventions do not take advantage of real-time *in vivo* neurochemical changes in dynamic brain processes such as disease progression and response to pharmacologic, cognitive, behavioral, and neuromodulation therapies. This is due in part to a lack of flexible research tools that allow *in vivo* measurement of the dynamic changes in brain chemistry. Here, we present a research platform, *WINCS Harmoni*, which can measure *in vivo* neurochemical activity simultaneously across multiple anatomical targets to study normal and pathologic brain function. In addition, *WINCS Harmoni* can provide real-time neurochemical feedback for closed-loop control of neurochemical levels via its synchronized stimulation and neurochemical sensing capabilities. We demonstrate these and other key features of this platform in non-human primate, swine, and rodent models of deep brain stimulation (DBS). Ultimately, systems like the one described here will improve our understanding of the dynamics of brain physiology in the context of neurologic disease and therapeutic interventions, which may lead to the development of precision medicine and personalized therapies for optimal therapeutic efficacy.

Normal brain function relies on a precise and delicate balance of chemical signaling mediated by the release of specific neurotransmitters. When this balance is disrupted by disease or injury, the results can be devastating. For example, decreased dopamine release is associated with anxiety, depression, cognitive impairment, fatigue, balance difficulties, and tremor^1^,^2^,^3^,^4^,^5^,^6^. Similarly, decreased levels of serotonin are associated with depression, anxiety, migraine headaches, impaired cognitive function, and insomnia^7^,^8^. These and other brain disorders affect up to one billion people worldwide^9^.

The last 30 years have seen a significant expansion of neuromodulation techniques that have become established forms of treatment for patients with certain brain disorders that do not respond to conventional pharmacologic, behavioral, and surgical interventions. These neuromodulation treatments typically involve electrical stimulation of anatomical targets linked to pathological neural activity associated with a specific disorder^10^. For example, electrical stimulation of deep brain structures, known as deep brain stimulation (DBS), has received approval from the United States Food and Drug Administration (FDA) for the treatment of advanced Parkinson’s disease (PD) and tremor^11^,^12^,^13^. Similarly, clinical DBS trials are underway in the United States (with CE Mark approval for clinical use in Europe) for the treatment of depression and epilepsy, with humanitarian device exemptions received for treatment of dystonia and obsessive-compulsive disorder^14^. Additionally, vagal nerve stimulation (VNS) has received FDA approval for treatment of pharmacoresistant epilepsy and major depressive disorder^15^.

Although the precise effects underlying therapeutic response remain a matter of debate, both clinical and preclinical studies suggest that proximal and distal changes in neural activity and neurotransmitter release are implicated in the neurobiology of disease^16^,^17^,^18^. For example, it has been suggested that transcranial magnetic stimulation (TMS) modulates γ-aminobutyric acid (GABA) activity^19^ mediating cortical excitability involved in Parkinson’s disease^20^, amyotrophic lateral sclerosis^21^, Tourette syndrome^22^ and dystonia^23^. Other studies suggest that VNS evokes norepinephrine release in the locus coeruleus, which may be involved in the therapeutic effects of neuromodulation for epilepsy^24^. DBS of the subthalamic nucleus (STN), a successful therapy for PD, has been associated with increased electrophysiological activity in neurons within the globus pallidus (GP)^25^,^26^,^27^ and substantia nigra^28^,^29^,^30^. Stimulation of the ventral capsule/ventral striatum and cingulate cortex for the treatment of major depressive disorder has been found to normalize neural activity in the anterior insula as well as the prefrontal, premotor, and dorsal anterior cingulate cortices^31^,^32^. DBS of the thalamic ventral intermedius (VIM) nucleus for treatment of tremor has been shown to change neural activation across the cerebello-thalamo-cortical circuit^33^,^34^. This change is associated with reduction of pathologic oscillations in cortical, thalamic, and cerebellar nuclei leading to tremor reduction in patients with essential tremor^35^,^36^. Interestingly, pre-clinical studies have shown that therapeutic neuromodulation strategies such as DBS of the STN, the medial forebrain bundle (MFB), and nucleus accumbens evoke the release of dopamine and adenosine in the striatum and nucleus accumbens, structures associated with control of motor and/or limbic behavior^18^,^37^. Most patients suffering from neurologic disease are treated with pharmacological agents that manipulate neurochemical changes, either as a primary intervention, or as an adjunct to other forms of therapy such as neuromodulation. Therefore, it is likely that the therapeutic effects of many clinical interventions may be mediated, at least in part, by proximal and distal changes in neurochemical activity^18^.

In order to measure neurochemical activity, a number of investigational devices have been previously developed (Table 1). However, important limitations with these devices, including but not limited to wired links to control computers, large physical dimensions, and lack of a built-in synchronized stimulation and recording system, have made neurochemical characterization of *in vivo* disease processes and therapeutic interventions, particularly in large animal models of behavior, a difficult endeavor. To begin addressing these limitations, we previously developed a portable wireless instantaneous neurotransmitter concentration sensing (*WINCS*) system^38^,^39^ that enabled high temporal resolution measurement of neurotransmitters and other electroactive molecules via fast scan cyclic voltammetry (FSCV). Unfortunately, its single recording channel limited its use for measurement of network changes in neurochemical activity. In addition, *WINCS* did not provide synchronized stimulation and neurochemical recording. Thus, characterization of stimulation-evoked neurochemical responses was difficult due to inconsistent delays between electrical stimulation and neurochemical measurements and, above all, the presence of stimulation artifacts superimposed on the neurochemical signals of interest. To address these limitations, we developed a next-generation research platform called *WINCS Harmoni*, designed for wireless multi-channel *in vivo* real-time measurement of neurochemical activity. *WINCS Harmoni* enables analysis and characterization of neurochemical activity, synchronization of neurochemical measurements and electrical stimulation, as well as real-time closed-loop control of neurochemical responses via titration of stimulation parameters and therapeutic optimization.

Here we report the results of a series of proof-of-principle experiments aimed at wireless measurement, characterization, and control of neurotransmitter release in healthy non-human primate (NHP), swine, and murine models of DBS using *WINCS Harmoni*. Specifically, we investigated its ability to simultaneously detect different neurochemical responses evoked by electrical stimulation. The study also validated the capability of *WINCS Harmoni* to measure stimulation-evoked dopaminergic responses across up to four recording channels and facilitate mathematical characterization of these responses as a function of stimulation parameters. Data were collected using carbon fiber microelectrodes (CFM) implanted within the medial-dorsal striatum of NHP, swine, and rodents. Finally, using a closed-loop paradigm, we tested an example application of the *WINCS Harmoni* platform for controlling striatal dopaminergic responses through automated modulation of DBS parameters in response to real-time changes in neurochemical activity *in vivo*.

## Results

### Simultaneous wireless control of stimulation, neurochemical recordings, and data telemetry

**Figure 1:**
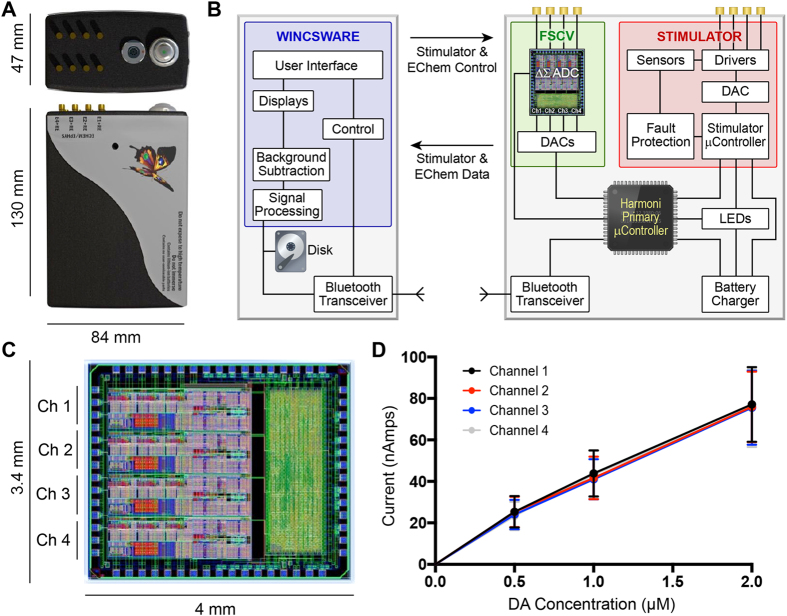
*WINCS Harmoni* device. (**A**) Photograph of the *WINCS Harmoni* device; (**B**) Schematic diagram showing the functional components in *WINCS Harmoni*, including the neurochemical sensing and stimulation boards, charging components, Bluetooth transceiver, primary microcontroller, and control software in the base station; (**C**) Image and dimensions of the *WINCS Harmoni* delta sigma (ΔΣ) analog-to-digital converter (ADC) that enables four channels of simultaneous neurochemical recordings; (**D**) Typical calibration curves for each of the four neurochemical recording channels in *WINCS Harmoni* showing a linear relationship between dopamine oxidation current and dopamine concentration.

*WINCS Harmoni* (Fig. 1) provided independent but synchronized stimulation and neurochemical measurements. Two separate rechargeable 6.5 watt-hour lithium-ion batteries in the *WINCS Harmoni* hardware provided up to 12 hours of continuous stimulation and 7 hours of continuous monitoring of neural activity on a single recording channel. The recording battery life was reduced to three hours when all four recording channels were used simultaneously. An external swappable battery allowed for an additional seven hours of continuous neurochemical detection for a single recording channel.

**Figure 2:**
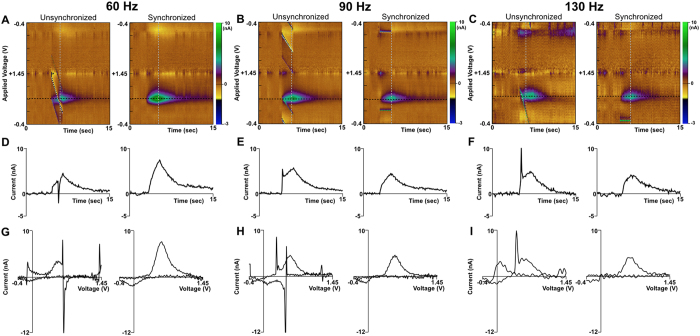
Effect of stimulation artifact with and without synchronization between neurochemical sensing and stimulation. (**A**–**C**) Dopamine detection of dopamine in a rodent undergoing medial forebrain bundle (MFB) deep brain stimulation (DBS) as shown by pseudocolor plots when a device without synchronization is used (left) versus *WINCS Harmoni* (right). The effect of unsynchronized stimulation artifacts is shown for 60 Hz (**A**), 90 Hz (**B**), and 130 Hz (**C**) stimulation frequencies are shown. (**D**–**F**) Time-series plot of FSCV data extracted at the dopamine oxidation potential (black dashed line in pseudocolor plot) for recorded dopamine signal without (left) and with (right) stimulation-neurochemical recording synchronization. Stimulations applied at 60 Hz (**D**), 90 Hz (**E**), and 130 Hz (**F**) are shown. (**G**–**I**) Voltammograms showing the effect of stimulation artifacts on the recorded dopamine signal recorded without (left) and with (right) stimulation-neurochemical recording synchronization for 60 Hz (**D**), 90 Hz (**E**), and 130 Hz (**F**) stimulation respectively. *WINCS Harmoni* is capable of controlling where the stimulation artifact appears on the voltammogram so it does not interfere with the target measurements as exhibited by artifact free time-series plots (**D**–**F**).

The integration of the hardware for stimulation and neurochemical recording into a single system enabled the interleaving of stimulation with FSCV recording. As a result, the impact of stimulation artifact on the measurement and characterization of low-amplitude FSCV responses was minimized by controlling where the stimulation artifact appears on the voltammogram, thereby ensuring any stimulation artifact present in the recorded data does not overlap with the signals of interest (Fig. 2). The physical dimensions of the device (84 mm × 130 mm × 47 mm) enabled neurochemical sensing and neurostimulation suitable for behavioral experiments in large animal models. Bench-top characterization tests showed that the stimulator was able to deliver biphasic charge-balanced pulses across up to four channels. Both *in vitro* and *in vivo* performance tests in NHP (n = 3), swine (n = 10), and rodents (n = 40) showed that the neurochemical recording module provided four independent channels of neurochemical detection with a signal acquisition bandwidth of 50 kHz and slew rates of up to 1200 V/s for the applied potential.

The Bluetooth digital communication between the remote *WINCS Harmoni* unit and the base-station computer running the WincsWare software allowed wireless device control and data telemetry for up to 10 meters. In addition, WincsWare allowed real-time visualization of filtered telemetry data from all recording channels (Figure S1), enabling on-line adjustments to the stimulation parameters, recording parameters (e.g., creation of arbitrary waveforms for detection of other neurochemicals), and electrode depth. Raw telemetry data was stored on the base station computer, which enabled both off-line and on-line signal processing, data analysis, and implementation of control strategies using third-party and custom-made software, such as MATLAB (The MathWorks, Inc., Natick, NJ), LabVIEW (National Instruments, Austin, TX), and Python.

### *In vivo* real-time measurement of neurochemical release

*WINCS Harmoni* was used to quantify the amplitudes of the neurochemical responses corresponding to the release of dopamine, serotonin, and adenosine as identified by their respective peak oxidation and reduction currents occurring at specific applied voltages with respect to a silver/silver-chloride (Ag/AgCl) reference electrode. With a standard triangular waveform^40^,^41^ ramping from −0.4 to +1.5 V, the oxidation current peaks occurred at approximately +0.6 V for dopamine, +0.6 V for serotonin, and +1.5 V (and a second oxidation peak at +1.0 V) for adenosine. The reduction peaks occurred at approximately −0.2 V for dopamine, −0.1 V (0.4 V) for serotonin, and +0.25 V (and a second peak at −0.05 V) for adenosine. For serotonin detection a N-shaped waveform is utilized (Figure S2) with an oxidation peak occurring at +0.6 V and a reduction peak at −0.1 V^42^.

**Figure 3:**
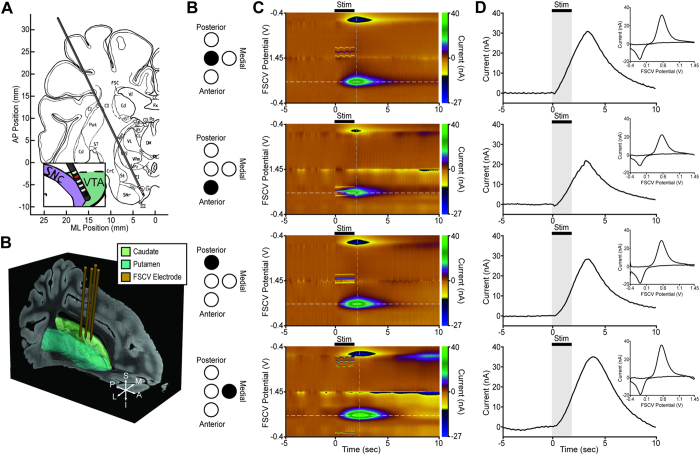
Representative multi-channel measurement of stimulation-evoked neurochemical responses in the swine brain. (**A**) A deep brain stimulation (DBS) electrode was implanted within the swine substantia nigra pars compacta/ventral tegmental area (SNc/VTA)^56^. Reprinted from Brain Research Bulletin, Vol. 49, Bernadette Félix, Marie-Eva Léger, Denise Albe-Fessard, J.-C Marcilloux,O Rampin, J.-P Laplace, A Duclos, F Fort, S Gougis, M Costa, N Duclos, Stereotaxic atlas of the pig brain, 73, 1999, with permission from Elsevier. (**B**) A representation of four carbon fiber microelectrodes (CFM) for neurochemical recording implanted within the swine striatum (green). The electrode position shown is from co-registration of the pre-operative targeting MRI with a 3D pig atlas^70^. (**C**) Schematic representation of the anatomical CFM location with respect to the target locations (**B**). (**D**) Pseudocolor plots showing dopamine detection via fast scan cyclic voltammetry (FSCV) from the four CFMs. Dopamine signals were measured using a standard pyramidal FSCV waveform in which the applied voltage was ramped from the holding potential of −0.4 V to the switching potential of +1.45 V and then back to −0.4 V at 400 V/s. (**E**) Time-series plots showing the current recorded at the dopamine oxidation peak (white dashed line in pseudocolor plot). For each CFM, the voltammogram showing the background subtracted stimulation-evoked dopamine signals recorded at the peak dopamine oxidation current (grey dashed line in the pseudocolor plot) is shown in the inset panel. Subtle differences in the dopamine current peak can be observed at the different electrode locations.

The *WINCS Harmoni* device measured simultaneous stimulation-evoked dopamine release across four CFMs implanted in the dorsal striatum of anesthetized swine (n = 12) during high frequency electrical stimulation of the substantia nigra pars compacta/ventral tegmental area (SNc/VTA) (Fig. 3). Maximal stimulation-evoked dopaminergic responses were recorded in the anterior lateral border of the caudate along a trajectory that typically ended along the lateral posterior border of the nucleus accumbens (Fig. 3A,B). Additionally, *WINCS Harmoni* detected spontaneous adenosine release in the striatum of 10 of these swine (Figure S3). Similarly, *WINCS Harmoni* measured dopamine release in the NHP striatum (n = 3) evoked by SNc/VTA stimulation (Figure S4A,B). Evoked dopamine was observed to increase with both stimulation amplitude and pulse width (Figure S4C,D).

**Figure 4:**
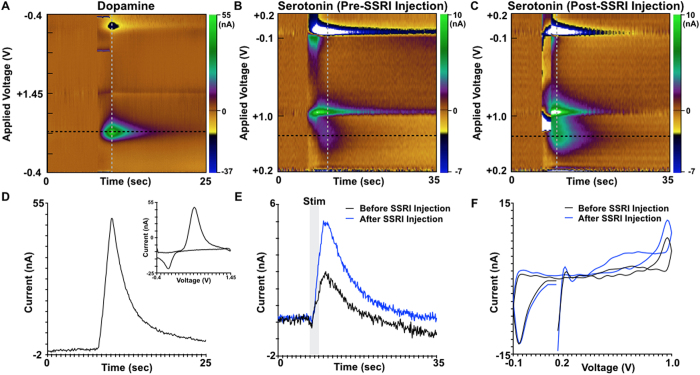
Recording of multiple neurotransmitters at two different anatomical targets in a rodent model of medial forebrain bundle deep brain stimulation. (**A**) Pseudocolor plot showing the detection of stimulation-evoked dopamine in the rodent striatum in response to medial forebrain bundle (MFB) stimulation. Dopamine measurement was performed using fast scan cyclic voltammetry (FSCV) and a standard pyramidal waveform from a −0.4 V holding potential to a +1.45 V switching potential. (**B**) Pseudocolor plot showing the simultaneous detection of serotonin in the substantia nigra pars reticulata (SNr) using FSCV with a standard N-shaped waveform from +0.2 V to +1.0 V, back down to −0.1 V, and up to +0.2 V. (**C**) Pseudocolor plot showing an increase in stimulation-evoked serotonin release in the SNr following systemic injection of the selective serotonin reuptake inhibitor (SSRI) fluoxetine. (**D**) Time-series plot showing the current recorded at the dopamine oxidation peak (black dashed line in pseudocolor plot). The inset panel shows the background subtracted voltammogram recorded at the peak dopamine oxidation current (grey dashed line in the pseudocolor plot). (**E**) Detection of evoked serotonin in the SNr before and after SSRI administration. Time-series data was extracted from the FSCV data at the black dashed line in panels (**B**) and (**C**). (**F**) Background subtracted cyclic voltammograms for serotonin before and after SSRI administration and extracted at the grey dashed line in panels (**B**) and (**C**) respectively.

In addition to the measurements in swine and NHP, *WINCS Harmoni* measured dopamine release in the rodent striatum (n = 40) evoked by MFB stimulation (Fig. 2). In one additional rodent, *WINCS Harmoni* measured simultaneous dopamine and serotonin release in the striatum and substantia nigra pars reticulata (SNr), respectively, evoked by MFB stimulation (Fig. 4). An additional oxidation was observed at the 1.0 V switching potential for the N-shaped waveform used to measure serotonin (Fig. 4C,D), similar to previously described SNr recordings^43^. *In vivo* measurements were compared to flow injection analysis (Figure S5). Immunohistochemistry was performed to confirm the location of the SNr recording electrode (Figure S6).

### Mathematical modeling of stimulation-evoked dopamine release

The dopaminergic system in all three animal models exhibited non-linear dopamine kinetics as a function of stimulation amplitude and pulse width. These dopaminergic data measured by *WINCS Harmoni* were characterized by parametric mathematical models of dopamine release, reuptake, dopamine transfer kinetics (including restricted diffusion), and adsorption/de-sorption at the electrode surface^44^. These models, trained on stimulation-evoked experimental dopamine responses, were able to accurately describe the non-linear relationships between DBS parameters and stimulation-evoked extracellular dopamine responses for 12 rodents (R^2^ = 0.90), 4 swine (R^2^ = 0.96), and 1 NHP (R^2^ = 0.96) in real-time (Figure S7). This is paramount as control of neurochemical release relies on a well-characterized and consistent relationship with stimulation parameters (e.g., stimulus intensity).

### Closed-loop control of stimulation-evoked striatal dopamine release

**Figure 5:**
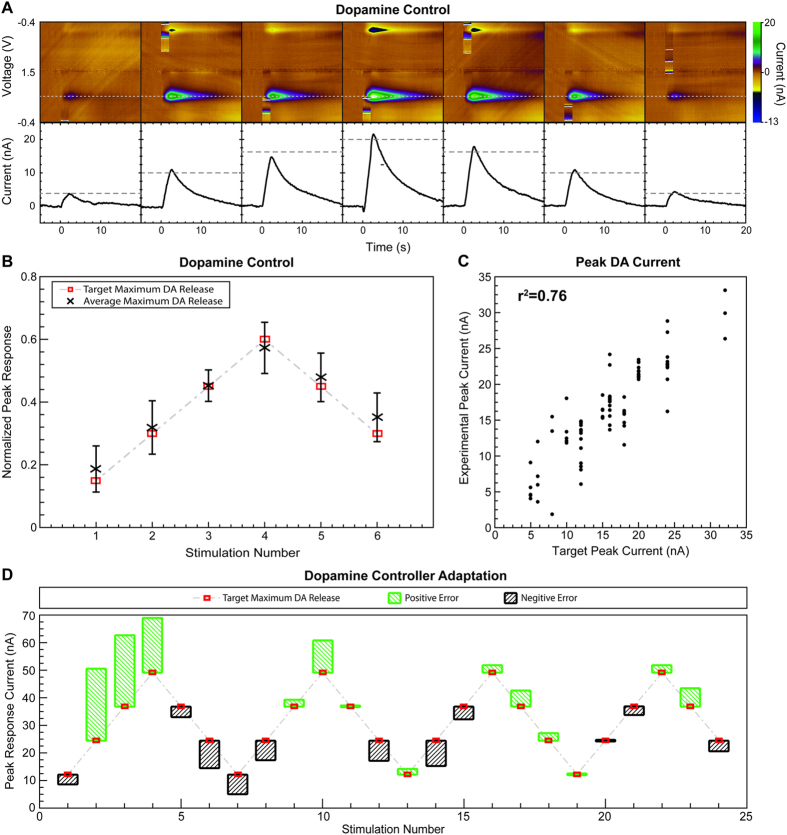
Closed-loop control of phasic dopamine release in a rodent model of medial forebrain bundle deep brain stimulation. (**A**) Pseudocolor plots (top) and dopamine oxidation current (bottom) showing control of stimulation-evoked dopamine response amplitude over seven dopamine target responses in one rodent model of medial forebrain bundle (MFB) deep brain stimulation (DBS). Pseudocolor plots (top) and time series measurements (bottom) show the dopamine oxidation current measured. A dashed grey line shows the target peak dopamine oxidation currents. Solid black lines show the kinetics of stimulation-evoked dopamine. (**B**) Dopamine release targets (red squares) and average experimentally evoked dopamine release (black x) evoked with stimulation parameters predicted by the artificial neural network (ANN) controller. Error bars represent standard deviation. Results from five rodents are normalized to the maximum response predicted by the ANN controller. (**C**) Regression analysis between the target dopamine responses and the peak dopamine responses evoked with parameters predicted by the ANN controller. Pearson’s correlation coefficient was r^2^ = 0.76. (**D**) Adaptation of the ANN controller when initiated with an incorrect calibration (n = 1). The error in the experimental response (green or black boxes) with respect to the target maximum dopamine release (red squares) decreased with each stimulation event as the ANN controller adapted.

The neurochemical control strategy based on artificial neural networks (ANN) tested in the rodent model of brain stimulation allowed control of the non-linear dopamine release (R^2^ = 0.79) by modulating stimulus amplitude and pulse width (Fig. 5A–C). Here, the trained ANN predicted stimulation parameters (i.e., pulse width and amplitude) required to evoke target dopamine responses. Figure 5A shows the raw FSCV data for seven consecutive stimulations in one rodent subject using parameters predicted by the control system. Each response evoked by MFB stimulation is shown in the form of a pseudocolor plot of the FSCV data (top). The pseudocolor plots depict dopamine oxidation (typically occurring at +0.6 V with respect to the Ag/AgCl reference) and reduction (typically at −0.2 V) peak responses. Each response is also shown by a time series (solid black lines, bottom) obtained at the dopamine oxidation voltage. A horizontal dashed gray line shows the target stimulation-evoked dopamine response for each of the seven stimulations. The controller error was determined by the difference between the target and the peak of each time series. As Fig. 5A shows, the experimentally-evoked responses followed the target dopamine responses with small errors. Figure 5B shows the ability of the controller to predict the stimulation parameters required to evoke a repeated pattern of six target peak dopamine responses (each with at least three repetitions) across five different animal subjects. Six target responses ranging from 15% to 60% of the maximal stimulation-evoked dopamine peak at the start of the experiment (typically between 35 and 80 nA) were selected as target maximum stimulation-evoked dopamine responses for testing the controller. Figure 5C shows a regression analysis demonstrating the ability of the controller to predict the stimulation parameters required to evoke the same target dopamine responses (R^2^ = 0.76) shown in Fig. 5B. Figure 5D shows one example of the ability of the control system to improve prediction error over time through adaptation of the ANN weights and biases. This adaptation allowed the control system to compensate for electrode drift, pH shifts, and other dynamic changes within the brain that affected the amplitude of the stimulation-evoked responses.

## Discussion

Surgical, pharmacologic, behavioral, cognitive, and neuromodulation therapies have been used to treat distinct neurologic diseases with mixed results. Clinical improvement has been limited at least in part by an incomplete understanding of how specific changes in neural network activity affect behavior. In order to achieve significant advances in patient treatment, the cascade of biomolecular effects that accompany normal and pathological behavior need to be elucidated. While research has shed light on the critical role of neurochemical signaling in the context of normal and pathological brain function, there remains much to understand about the effects of changes in neurochemical signaling in response to disease progression or to treatment. The present study explored the potential for using *WINCS Harmoni* to address a gap in the understanding of normal and pathologic neurophysiology by analyzing neuromodulation-induced changes in neurochemical dynamics. *WINCS Harmoni* is an investigational platform for wireless multichannel neurochemical sensing and synchronized stimulation that leverages technology developed for the Wireless Instantaneous Neurotransmitter Concentration Sensing (*WINCS*) system and the Mayo Investigational Neuromodulation Control System (MINCS)^38^,^39^,^45^. Here, *WINCS Harmoni* was used to probe neural networks by activating one node within a neural circuit and measuring the downstream neurochemical effects of electrical stimulation. It is important to note that this study was not designed to address neurochemical activity in the context of disease, but rather as a first step toward achieving that goal by testing the capabilities of *WINCS Harmoni* in healthy animal models of DBS. *WINCS Harmoni* is based on technologies that can be scaled toward implantable systems. Signal amplification, acquisition, filtering, and digitization is performed within the WINCS *Harmoni* unit. As such, *WINCS Harmoni* provides a unique research platform capable of collecting, integrating, and analyzing multi-modality data to study and modulate neural activity in the context of specific disease types, symptoms, and therapeutic interventions, and could be used as an adjunct to drug delivery and neuromodulation therapies to optimize therapeutic effects while minimizing side effects.

Understanding the normal and pathological physiology underlying brain function will require the use of different analysis modalities (e.g., neurochemical sensing, electrophysiological monitoring, functional imaging) to study neural activity across distinct brain regions and the interactions between them. *WINCS Harmoni* is able to synchronize multiple channels of neurochemical recording with other forms of analysis or therapies (in this case electrical stimulation), facilitating the study of neural activity by ensuring that stimulation artifacts typically observed in *in vivo* stimulation-evoked neurochemical recordings do not overlap with the signals of interest. Additionally, *WINCS Harmoni* provides a platform for wireless control and telemetry, thus allowing for seamless real-time interfacing with data analysis software to enable characterization of neurochemical responses. Our results demonstrate that *WINCS Harmoni* is suitable for detecting changes in neurochemical signaling across different animal models. For this study we focused on dopamine in the dorsal striatum of rodents, swine, and NHP, but the applicability of this device can be generalized to other neurotransmitters/neurochemicals, brain targets, and animal models. For example, *WINCS Harmoni* was able to simultaneously measure stimulation-evoked dopamine release in the dorsal striatum and serotonin release in the SNr of rodents (see Fig. 4). Additionally, an oxidation signal was observed at the 1.0 V switching potential of the SNr recording (Fig. 4B,C). This signal, agrees with previously characterized simultaneous serotonin and histamine release in the rat SNr following medial forebrain bundle stimulation described by Hashemi *et al*.^43^.

The high spatial and temporal resolution of FSCV at a CFMs^40^,^46^,^47^, coupled with the ability of *WINCS Harmoni* and WincsWare to wirelessly interface in real-time to data analysis software such as MATLAB allowed the measurement and characterization of proximal and distal neurochemical activity at multiple time scales (in the order of milliseconds to minutes) acquired under non-restrictive experimental paradigms. This is important because different diseases and symptoms respond to different therapies on different timescales, which can vary from seconds to months^48^,^49^,^50^,^51^,^52^. For example, fast therapeutic responses, such as those found in DBS for treatment of tremor, may be mediated by nearly instantaneous and rapidly reversible mechanisms involving immediate modulation of pathological neural activity^53^. Slow therapeutic responses, such as those observed in DBS for treatment of dystonia and psychiatric conditions may be mediated, at least in part, by changes brought about through synaptic plasticity and anatomical reorganization^54^. These different response times suggest that neuromodulation and other forms of therapy act through different mechanisms across different diseases, disease states, and anatomical targets^52^, likely mediated by short- and long-term changes in neurochemical activity. Additionally, sustaining long-term optimal therapeutic benefits may require frequent adjustments to the therapeutic paradigms (e.g., neuromodulation parameters, medication dose, etc.) in order to accommodate the dynamic neurochemical environment. Thus, therapeutic paradigms in the form of closed-loop interventions that can adapt in response to changes in neural activity will likely improve clinical outcomes and reduce the frequency of outpatient visits. *WINCS Harmoni* enables the development and implementation of such closed-loop therapies by responding to neurochemical feedback, potentially improving the therapeutic effect by modulating neurochemical levels with high precision compared to standard drug delivery techniques.

In summary, *WINCS Harmoni* can be used to monitor dopaminergic activity, enable kinetic characterization of stimulation-evoked dopamine (via mathematical models), and automatically adjust stimulation parameters (via artificial neural networks) to achieve target dopaminergic responses. These capabilities can be leveraged to develop adaptive therapies that can compensate for both sudden, as well as slow, physiological changes taking place as a consequence of the condition that is being treated. As demonstrated here, *WINCS Harmoni* was able to take advantage of closed-loop strategies to control stimulation using real-time *in vivo* measurement and characterization of neurochemical activity as a feedback signal. We focused our efforts on real-time measurement, characterization, and control of stimulation-evoked dopamine in healthy anesthetized rodents, swine, and NHP. However, future studies using this platform should focus on characterizing cell types (as a function of their neurotransmitters) and differences in neurochemical dynamics in the context of disease state and therapeutic efficacy across different diseases, anatomical targets, and therapeutic interventions. In turn, the results of such studies will further our understanding of the therapeutic mechanisms of neuromodulatory and pharmacologic interventions, ultimately leading to the development of personalized therapies for sustained therapeutic efficacy.

## Materials and Methods

### Hardware and software for simultaneous stimulation, neurochemical measurement, and data analysis

*WINCS Harmoni* is a device capable of detecting neurochemical activity at four independent sensors and concurrently applies stimulation to any combination of four electrode contacts. Stimulation can be synchronized with up to four channels of FSCV to control where stimulation artifacts appear in the recorded data and ensure these do not obscure the neurochemical measurements of interest. The *WINCS Harmoni* electronics are implemented on two multilayer printed circuit boards (PCBs), one for FSCV, control and telemetry, and one for stimulation. Each PCB is powered by its own rechargeable 6.5 watt-hour lithium-ion battery (UBP001, Ultralife Corp., Newark NY) to isolate the electrochemistry and stimulation systems.

A custom analog-to-digital converter (ADC) integrated circuit (Mayo Clinic Special Purpose Processor Development Group, Rochester, Minnesota) allows multi-channel FSCV using custom-designed FSCV voltage waveforms for each channel. Each of the four channels incorporates a three-stage continuous-time delta-sigma (ΔΣ) modulator that produces a digital bit stream at 12.8 Mb per second. A decimation filter in the ΔΣ ADC resolves the digital bit stream into 16-bit samples that are delivered to a microcontroller (LPC1769, NXP Semiconductors, Eindhoven, Netherlands) and thence to a Bluetooth radio (WT41, Bluegiga Technologies, Inc., Espoo, Finland) at a rate of 100,000 samples per second. Wireless data telemetry takes place on the 2.4 GHz frequency band approved by the Federal Communications Commission (FCC).

The stimulator PCB has a separate microcontroller that controls a 16-bit digital-to-analog converter (DAC) (AD5541A, Analog Devices, Inc., Norwood MA) to deliver stimulus pulse sequences. Each stimulus can either be monophasic or biphasic, voltage- or current-regulated, and can be steered to any combination of four electrode contacts. The stimulus amplitude in voltage-regulated mode can be adjusted between 50 mV and 10 V, in 10 mV steps. In current-regulated mode, stimulation may be adjusted between 10 μA and 10 mA, in 10 μA steps. Pulse duration can range between 50 μs and 2 ms. Redundant fault detection circuits, including circuits that measure charge delivered per pulse, protect the subject from unsafe stimulation. Synchronization of stimulation with FSCV acquisition is achieved by specifying a delay (in milliseconds) between the start of each FSCV acquisition and the onset of the first pulse in a stimulus train. The optimal delay for synchronization must be selected based on the desired stimulation parameters and FSCV waveform(s).

The *WINCS Harmoni* hardware is controlled by a custom software application, WincsWare, running on a Windows PC (Figure S1). Developed in Microsoft^®^ Visual C# (Microsoft Co., Redmond, WA), WincsWare provides a highly configurable user interface for real-time control of stimulation, FSCV, and data visualization. The measured neurochemical data are transmitted wirelessly, via Bluetooth, to a base station Windows^®^ (Microsoft Co., Redmond, WA) computer running WincsWare. The base station provides real-time visualization of filtered telemetry data and storage of raw telemetry data. An application program interface enables applications such as MATLAB to perform on-line data analysis and stimulation control.

### Animal subjects

Stimulation-evoked neurochemical responses were measured by surgically implanting DBS electrodes and CFM in 40 female Sprague-Dawley rats weighing 250–350 g, 12 male young adult pigs age 8–12 months weighing 25–35 kg, and 3 male rhesus macaque NHP age 36 months weighing 11–13 kg. Closed-loop control of stimulation-evoked dopamine levels was performed in 12 female Sprague-Dawley rats. All animal studies were performed *in vivo* with approval of the Institutional Animal Care and Use Committee (IACUC) and following National Institutes of Health (NIH) guidelines for animal research. Rodents were kept in social housing; swine were kept in individual areas. All animals were kept on a 12 hour light/dark cycle at a constant temperature (21 °C) and humidity (45%) and were fed once daily, with ad libitum access to water.

### Surgical procedure

Prior to surgery, rodents were sedated with intraperitoneal urethane (1.5 g/kg in a 0.26 g/mL saline solution). Swine were sedated with intramuscular Telazol (6 mg/kg) and xylazine (2 mg/kg), followed by intubation and inhalation anesthesia (1.5–3% isoflurane during electrode implantation and 1.5–2% isoflurane during the rest of the experiment). NHP were sedated with intramuscular ketamine (10 mg/kg) and xylazine (0.5 mg/kg), followed by intubation and inhalation anesthesia. Analgesia was maintained with intramuscular (IM) buprenorphine (0.06 mg/kg for rodents, 0.03 mg/kg for swine) or slow release (SR) buprenorphine (0.06 mg/kg for NHP). A scalp incision (1.5–2.0 cm for rodents, 5–7 cm for swine and NHP) was made to expose the skull, and three trephine burr holes (approximately 3 and 7 mm in diameter for rodent and large animals, respectively) were drilled to allow implantation of stimulating, neurochemical recording, and reference electrodes. A 3 mg intravenous (IV) bolus followed by a 17 mg/hr intravenous drip of vecuronium bromide was administered to swine and NHP during neurochemical recording to prevent stimulation-induced motion artifacts. For swine and NHP, core body temperature (36–38 °C), heart rate (70–110 bpm for swine, 100–130 bpm for NHP), blood pressure (110/80 mmHg for swine, 98 ± 17/54 ± 13 mm Hg for NHP), and blood-oxygen levels (SpO2 of 100%) were continuously monitored to assess the depth of anesthesia and ensure animal welfare.

### Stereotactic targeting and electrode implantation

Anesthetized animals were secured in a stereotactic headframe (David Kopf Instruments, Tujunga, CA) for locating the anatomic targets for the DBS and the neurochemical recording electrodes. For rodents, stainless steel bipolar electrodes (250 μm in diameter) with 20 mm long polyimide-coated shaft and a 1000 μm exposed tip (PlasticsOne, Roanoke, VA) were used for stimulation. The stimulating electrodes were implanted into the rodent MFB using a dual micromanipulator system (David Kopf Instruments, Tujunga, CA). The same stereotactic system was used to implant a custom-made CFM into the rat striatum for neurochemical recording via FSCV. The stereotactic coordinates of both the rodent MFB and striatum (Table S1) were obtained relative to bregma according to the Paxinos and Watson atlas of the rat brain^55^. Neurochemical recording electrodes had an exposed carbon fiber tip, 7 μm in diameter and 50–100 μm long. A Ag/AgCl electrode was implanted into the parenchyma of the contralateral brain hemisphere as a reference for the neurochemical recordings.

Swine and NHP were placed in a custom-made stereotactic headframe (Shon *et al*., 2010) for MRI-based targeting of the SNc/VTA, STN, and striatal targets. The stereotactic targets and electrode trajectories for swine and NHP were defined using the COMPASS stereotactic targeting platform (COMPASS International Innovations, Rochester, MN) and an anatomical atlas of the swine brain^56^ and of the NHP brain^57^. A six-contact DBS electrode, 0.625 mm in diameter, and 0.5 mm contact height with a 0.5 mm inter-contact spacing (NuMed, Hopkinton, NY) was implanted into the SNc/VTA region of swine and STN region of NHP. One NHP was implanted with an additional DBS electrode in the SNc/VTA. Electrode insertion for both DBS and the neurochemical recording electrodes was performed using a computer-controlled microdrive (Alpha Omega Co., Alpharetta, GA).

### Measurement of stimulation-evoked neurochemical responses

Subsequent to stereotactic targeting and electrode implantation, the target region (MFB for rodents and SNc/VTA for swine and NHP) was stimulated using a comprehensive range of stimulation parameters (Table S2). Simultaneously, *WINCS Harmoni* measured the magnitude of stimulation-evoked dopamine release at the CFM. Neurochemical detection of dopamine was performed by first holding the electric potential at the CFM at −0.4 V with respect to the Ag/AgCl reference electrode. Next, the CFM potential was linearly increased to a +1.4 V potential, also known as the “switching potential”, which is higher than the oxidation potential of dopamine. Finally, the CFM potential was linearly decreased back to the holding potential. The potential sweep was performed a rate of 400 V/s and repeated at a frequency of 10 Hz^40^,^58^,^59^. The CFM was held at the resting potential between scans^60^. Dopamine at the surface of the electrode was oxidized to form dopamine-o-quinone during the positive voltage sweep. The resulting dopamine-o-quinone was then reduced back to dopamine during the negative sweep. FSCV measured the electrical current resulting from the electron transfer, which takes place during these redox reactions between the oxidized and reduced molecules and the microelectrode. Dopamine oxidation currents proportional to the number of molecules undergoing redox reactions allowed estimation of dopamine concentrations using pre-operative and post-operative *in vitro* calibration of each CFM through flow injection analysis (Fig. 1D)^42^. The presence of dopamine was confirmed in a small cohort by administering the dopamine reuptake inhibitor nomifensine (20 mg/kg i.p. as the hydrochloride salt; Sigma, St. Louis, MO)^61^. The presence of serotonin, detected using an N-waveform^42^, was confirmed by administering the selective serotonin reuptake inhibitor fluoxetine (20 mg/kg i.p. as the hydrochloride salt; Sigma, St. Louis, MO)^43^.

### Multi-channel detection of stimulation-evoked neurochemical responses

To investigate the ability of *WINCS Harmoni* to conduct quasi-simultaneous neurochemical recording across multiple channels within the same target, as well as within different targets, four parallel CFM were implanted into the swine striatum (n = 10). Electrodes were held in place using a Leksell stereotactic head frame and a “Ben Gun” concentric electrode holder^62^,^63^. Electrodes were separated by 2 mm, with one electrode placed on the MRI-identified target for neurochemical recording, one electrode medial to the target trajectory, one electrode lateral, one electrode anterior, and one electrode posterior (Fig. 3). Electrode depth for all recording microelectrodes was controlled by the computer-controlled microdrive or micromanipulator. One rodent was implanted with two neurochemical recording electrodes. The first electrode was implanted in the striatum and the second in the SNr for simultaneous detection of striatal dopamine and SNr serotonin. Stimulation parameters were identical for both multi-channel and single-channel FSCV recordings. FSCV parameters for multi-channel recordings were also identical, except in the case of serotonin recordings, for which an N-waveform was used instead of a triangular waveform^43^.

### Calibration of carbon fiber microelectrodes for neurochemical recording

Redox currents measured during FSCV scans were plotted against the applied potential to generate cyclic voltammograms (Figs 2D–F and 4F for dopamine and serotonin respectively). The cyclic voltammograms were used to identify the unique signatures for each neurotransmitter and distinguish it from other electroactive analytes. Calibration curves defining the relationships between dopamine concentration and corresponding oxidation currents measured experimentally were obtained both pre-operatively and post-operatively using flow injection analysis. Each CFM was placed in a flowing stream of Tris buffer solution while multiple five second boluses of a Tris buffered dopamine solution (e.g., 0.1 μM, 0.5 μM, 1.0 μM) were injected via an electronic loop injector at 6 mL/min. Flow control was performed using a FIAlab 3200 injection system (FIAlab Instruments, Seattle, WA).

### Parametric characterization of stimulation-evoked dopaminergic kinetics

The ability of *WINCS Harmoni* and WincsWare to implement data analysis and control techniques via the MATLAB API was investigated in rodent, swine, and NHP models of DBS by characterizing the stimulation-evoked dopaminergic responses using mathematical models^44^. For this analysis, stimulation amplitudes and pulse widths leading to the minimal detection (i.e., threshold) and/or saturation of evoked dopamine responses were identified for each subject and used to determine a comprehensive range of stimulation parameters. Stimulation parameters were selected from within this range and applied in random order for either 0.5 or 2.0 seconds to capture system dynamics while eliminating the effects of hysteresis. Each stimulation event was followed by a dopamine stabilization period of either 30 or 300 seconds, depending on the duration of each stimulation event. Stimulation-evoked dopamine responses were first filtered with a 1 KHz third order Butterworth zero-phase shift filter to remove high-frequency noise, and then de-trended (i.e., removal of background currents) to eliminate the effects of capacitive background currents^64^ (Figure S8A). Next, the stimulation-evoked responses were fit using mathematical compartmental models of release, reuptake, transfer kinetics (e.g., restricted diffusion), and adsorption/de-sorption^65^,^66^,^67^ (Figure S8B,C). The parameters of the models were used to describe the kinetics of dopamine responses as a function of the stimulation parameters.

### Real-time feedback control of stimulation-evoked striatal dopamine

The feasibility of using *WINCS Harmoni* to provide real-time feedback control of striatal dopamine responses was evaluated experimentally in the rodent and swine models of DBS. Here, a strategy to control stimulation-evoked dopaminergic responses was implemented using artificial neural networks (ANN) due to their ability to model dynamic non-linear systems from experimental input/output data^68^. This predictive model of stimulation-evoked dopaminergic release characterized the inverse relationship between stimulation parameters and evoked dopamine levels by determining the stimulation parameters required to evoke and sustain target *in vivo* stimulation-evoked striatal dopamine responses (Figure S9). Details on the ANN architecture and training algorithms are presented elsewhere^44^. Briefly, the predictive controller consisted of a double layer ANN with sigmoidal and linear transfer functions in the input and hidden layers, respectively. The ANN-based controllers had two inputs, two outputs, and seven to nine hidden neurons mapping the relationship between inputs and outputs were used. The inputs to the predictive model corresponded to two mathematical model parameters specifying changes in dopamine concentration. The outputs of the predictive model were the stimulation pulse width and amplitude required to achieve the desired dopaminergic response specified by the inputs. The number of neurons in the hidden layer was optimized for each data set. The weights of the stimulation predictor were adjusted using an adaptive strategy based on the gradient descent method^69^ to minimize the mean squared error between the expected neurochemical responses and the actual stimulation-evoked neurochemical responses. These ANN controllers were designed, trained, and evaluated in MATLAB, and interfaced to *WINCS Harmoni* via an application program interface (API) that allowed real-time bi-directional communication between MATLAB and the *WINCS Harmoni* software, WincsWare.

The controller was evaluated under two testing paradigms. First, a constant peak dopamine response was targeted (Fig. 5A). Target dopamine values were selected by identifying a mid-range dopamine oxidation current between activation and saturation thresholds. Second, regulation of stimulation-evoked dopamine levels between two user-set thresholds of 15% and 60% of the maximal stimulation-evoked dopamine peak was targeted (Fig. 5B). Performance of the feedback controller was evaluated using the mean squared error (MSE) between the desired stimulation-evoked striatal dopamine responses and the experimental responses measured when the DBS target (SNc/VTA or MFB) was stimulated using controller-predicted parameters.

### Tissue Preparation and Immunohistochemistry

Recording and stimulating electrode tip positions were identified histologically (Figure S6). Prior to perfusion, electrical lesions (1 mA direct current for 10 seconds) were induced using an optical isolator and programmable pulse generator (Iso-Flex/Master-8; AMPI, Jerusalem, Israel). Rats were perfused with 0.9% saline followed by 4% paraformaldehyde in 0.1 M phosphate buffer (PB). The brains were removed and post-fixed in 4% paraformaldehyde overnight, stored into 25% glycerol in 0.1 M PB solution overnight for cyroprotection. Th e tissue was then blocked, frozen in dry ice and cut in the coronal plane on a sliding microtome. Frozen coronal sections (40 μm thick) were stored in a solution of 0.1 M NaKPO4 and 0.4% Na Azide until use.

Sections of interest were mounted sequentially onto subbed slides and allowed to dry overnight. The slides were then rehydrated in xylenes and a graded series of ethanol diluted in distilled water (100%, 95%, 70%; 1 × 2 minutes each), and then placed into cresyl violet solution for 3 minutes. Sections were rinsed in ascending concentrations of ethanol (25%, 70%; 1 × 4 minutes: one rinse, four minutes per rinse) to remove the excess stain, before placing into a cresyl differentiator (absolute alcohol and glacial acetic acid mixture) for 2 minutes. Afterwards, the sections were dehydrated by passing them through ascending alcohol (95%, 1 × 2 minutes; 100%, 2 × 2 minutes) washes and a set of xylenes washes (3 × 5 minutes). Following dehydration, slides were coverslipped in Eukitt mounting medium (Thermo Fisher Scientific, Waltham, MA).

For immunohistochemical analysis, brain sections were rinsed 3 × 2 minutes in 0.01 M phosphate buffered saline (PBS) containing 0.2% Triton X-1000 (Tx) and pre-treated with 3% hydrogen peroxide in PBS-Tx for 10 minutes. The sections were then rinsed 3 × 2 minutes in PBS-Tx, and then blocked in 10% normal goat serum (Gibco, Thermo Fisher Scientific) for one hour. The sections were incubated with primary antibody solution containing mouse anti-TH (1:4000, Millipore AB152) in 10% normal goat serum overnight at 4 °C with gentle shaking.

Following primary incubation, sections were rinsed 3 × 2 minutes in PBS-Tx, incubated in anti-rabbit secondary antibody coupled to biotin (BA-1000; Vector Laboratories, Burlingame, CA) for one hour, and then incubated for Avidin-Biotin Complex (ABC) amplification (ABC Standard Kit; Vector Laboratories) for an additional hour after a set of PBS-Tx rinses. Following amplification, sections were rinsed for 3 × 2 minutes in 0.1 M PB, and then placed into DAB HRP substrate (Vector Laboratories) for antigen-antibody compound detection. After a last set of 0.1 M PB rinses for 3 × 2 minutes, sections were mounted onto subbed slides and allowed to dry overnight at room temperature. The slides were then dehydrated in a series of ascending ethanol solutions diluted in distilled H2O (70%, 95%, 1 × 2 minutes each; 100%, 2 × 2 minutes) and a set of xylene washes (3 × 5 minutes), and then coverslipped with Eukitt mounting medium in preparation for image analysis.

### Imaging Analysis

Coronal brain sections were imaged on a Leica DMR microscope (Wetzlar, Germany) with an Olympus DP70 digital camera (Shinjuku, Tokyo, Japan). Each coronal brain section was imaged using a 10x objective, then stitched together using Adobe Photoshop CC15 (Adobe, San Jose, CA). The corresponding anterior-posterior levels of the coronal brain slices were identified using the Paxinos and Watson brain atlas^55^.

## Additional Information

**How to cite this article:** Lee, K. H. *et al*. WINCS Harmoni: Closed-loop dynamic neurochemical control of therapeutic interventions. *Sci. Rep.* **7**, 46675; doi: 10.1038/srep46675 (2017).

**Publisher's note:** Springer Nature remains neutral with regard to jurisdictional claims in published maps and institutional affiliations.

## Supplementary Material

Supplementary Information

## Figures and Tables

**Table 1:** A comparison of commonly available devices capable of FSCV.

	*WINCS Harmoni (This Work*)	*WINCS*^42^,^71^	*UEI (Universal Electrochemical Instrument*)^72^,^73^	*P. Phillips*’ *FSCV System*^74^,^75^	*Pinnacle FSCV System (wireless system*)^76^	*WaveNeuro FSCV System*^77^	*Reference 600+*^78^	*EZstat-Pro*^79^
*Organization*	*Mayo Clinic*	*Mayo Clinic*	*University of North Carolina*	*University of Washington*	*Pinnacle*	*Pine Instruments*	*Gamry*	*NuVant Systems*
*Number of FSCV channels*	4	1	4	4	1	1	1	1
*Wireless capabilities*	yes	yes	no	no	yes	no	no	no
*Built in synchronized stimulator*	yes	no	no	no	no	no	no	no
*Voltage range*	2 V – adjustable center	2.5 V – adjustable center	±10 V	±10 V	−0.6 V to + 1.5 V	± 3.3 V	± 12 V	± 10 V
*Maximum linear scan rate*	1200 V/s	1000 V/s	> 10,000 V/s	500 V/s	1000 V/s	5000 V/s	1500 V/s	500 V/s
*Current range*	± 1 μA, ± 2 μA, ± 3 μA, ± 4 μA, ± 5 μA, ± 6 μA, ± 7 μA, ± 8 μA	± 1.25 μA	2 μA default - defined by headstage	1.22 μA	5.6 μA, 50.6 μA	2 μA default - defined by headstage	11 ranges (60 pA–600 mA)	1 μA, 100 μA, 10 mA, 1 A
*DAC resolution*	16-bit	12-bit	16-bit	12-bit	18-bit	16-bit	16-bit	16-bit
*DAC update rate*	100 kS/s	100 kS/s	2 MS/s	1 MS/s	1000 S/voltammogram	2 MS/s	10 MS/s	250 kS/s aggregate (125 kS/s per channel)
*Analysis software*	WincsWare	WincsWare	HDCV	HDCV, TarHeel CV, Demon	Pinnacle FSCV software	HDCV	Framework	EZWare
*Real-time pseudocolor plot display*	yes	yes	yes	no	yes	yes	no	no
*Real-time analysis and control*	yes (via Application Program Interface)	no	no	no	no	no	no	no
*Use in Clinical Studies*	FDA physiologic device exception for OCD	Yes^80^,^81^,^82^	no	Yes^83^,^84^	no	no	no	no
