# Supplementary material for: WINCS Harmoni: Closed-loop dynamic neurochemical control of therapeutic interventions
Source: Sci Rep. 2017 Apr 28;7:46675. doi: 10.1038/srep46675 (PMC5408229; doi:10.1038/srep46675)
Supplement: Supplementary Information [file srep46675-s1.pdf]

Manuscript SREP-16-27416

Title: WINCS Harmoni: Closed-loop dynamic neurochemical control of therapeutic interventions.

Authors:

Kendall H. Lee[1,2,3,*], J. Luis Lujan[1,2,*], James K. Trevathan[4], Erika K. Ross[1], John J. Bartoletta[1], Hyung Ook Park[1], Brian S. Paek[4], Evan Nicolai[1], Jannifer H. Lee[1], Paul H. Min[1,2], Christopher J. Kimble[5], Charles D. Blaha[1†], Kevin E. Bennet[1,5]

Affiliations:

[1] Department of Neurologic Surgery, Mayo Clinic, Rochester, MN 55905
[2] Department of Physiology and Biomedical Engineering, Mayo Clinic, Rochester, MN 55905
[3] Department of Physical Medicine and Rehabilitation, Mayo Clinic, Rochester, MN 55905
[4] Mayo Graduate School, Mayo Clinic, Rochester, MN 55905
[5] Division of Engineering, Mayo Clinic, Rochester, MN 55905
[*] These authors contributed equally.
† Corresponding author.

SUPPLEMENTARY MATERIALS – SREP-16-27416

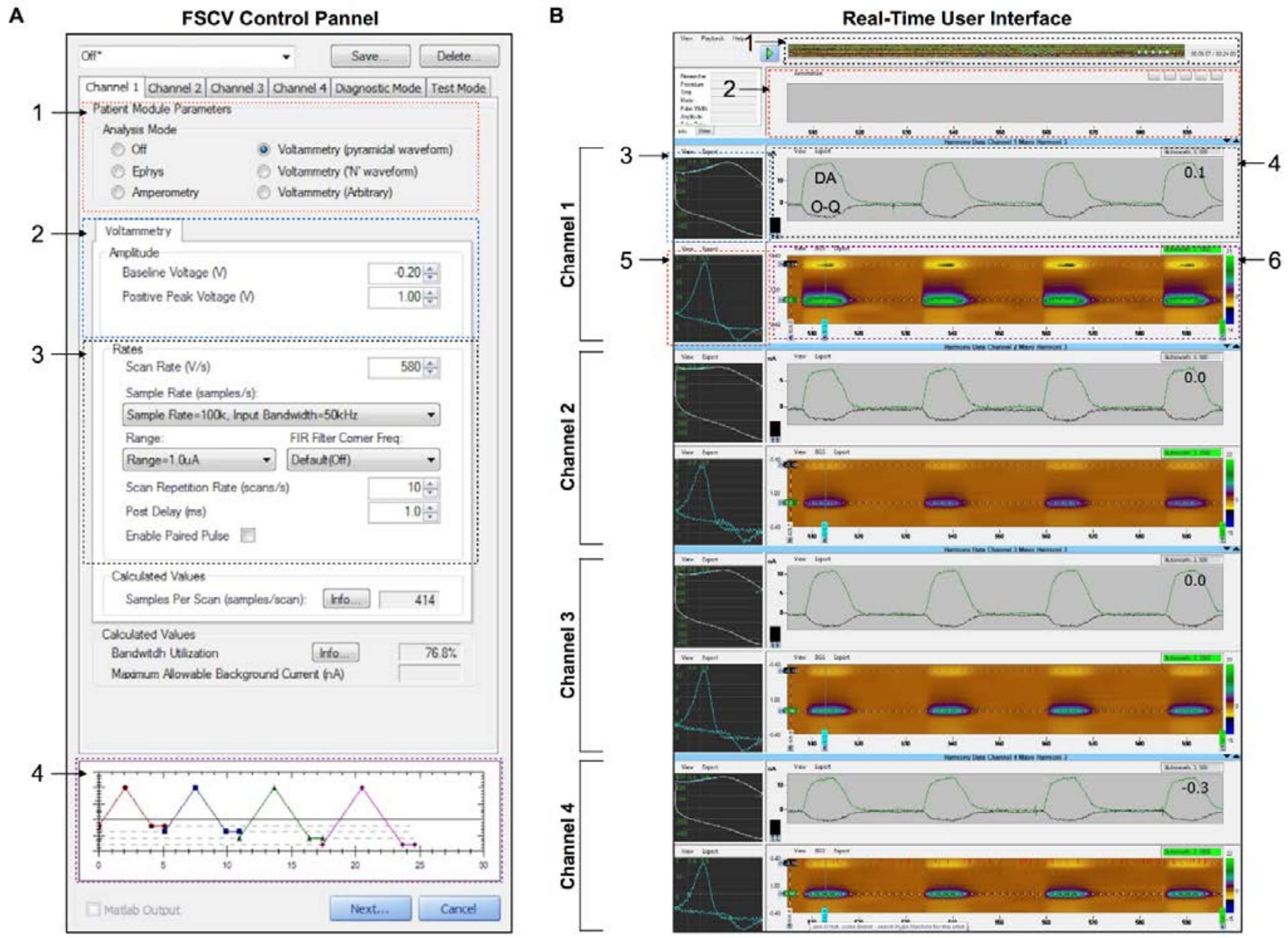

Figure S1. Graphical user interface of *WINCS Harmoni* software: WincsWare. (**A**) Module for real-time on line or queued adjustment of sensing parameters. This user interface allows the selection of fast scan cyclic voltammetry (FSCV) waveform or the selection of other measurement techniques (e.g., amperometry or electrophysiology) (**A1**). It also allows the selection of holding and switching potentials (**A2**), scan rate, sample rate, and range (**A3**), and provides schematic feedback of the effects of the parameters selected (**A4**). (**B**) The graphical user interface provides real-time visualization of the recorded data for all four-channels. Data shown corresponds to *in vitro* dopamine measurements using a flow injection system. The visual display includes a pseudocolor plot depicting data acquired throughout a recording session (**B1**), a panel for user annotations (**B2**), voltammograms (measured current as a function of applied potential) (**B3**), a time series of the measured current (**B4**), background-subtracted voltammograms (dopamine [DA] oxidation peaks can be observed at: ~+0.6 V and dopamine o-quinone [O-Q] reduction peaks at ~ -0.2 V) (**B5**), and pseudocolor plot of electrode current amplitude (intensity) as a function of applied potential (vertical axis) over time (horizontal axis) (**B6**).

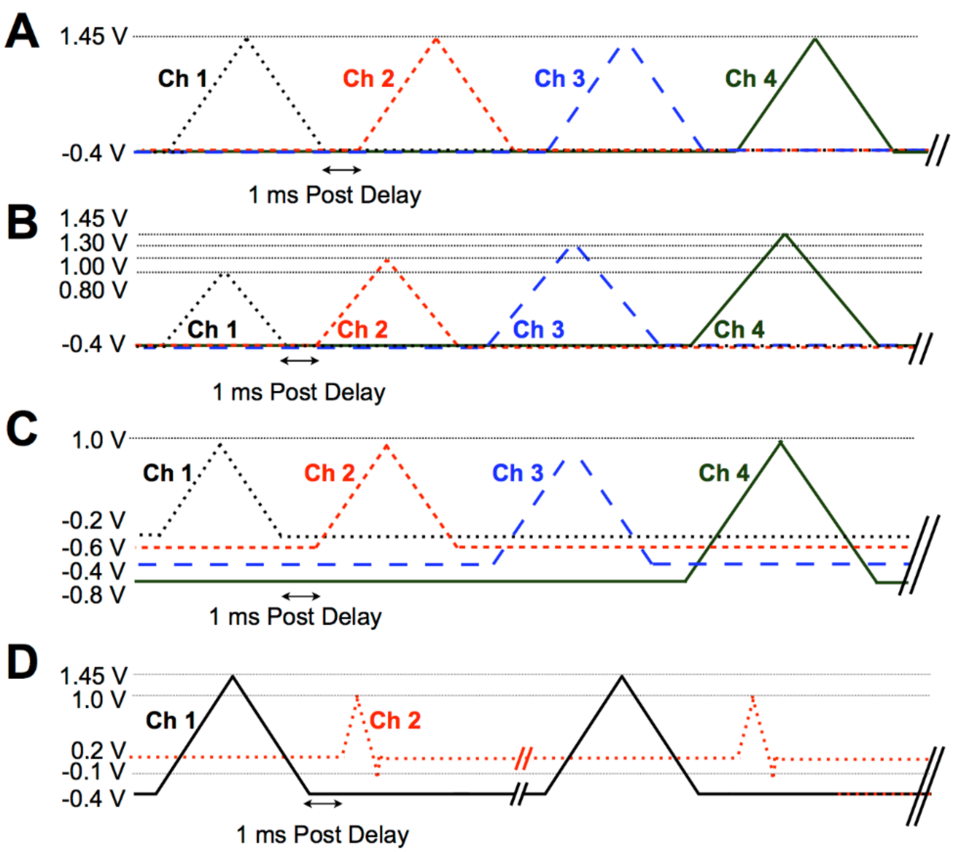

Figure S2. Parameters for fast scan cyclic voltammetry (FSCV) **detection of distinct neurochemicals at multiple recording channels.** (**A**) *WINCS Harmoni* provides up to four independent channels for FSCV recordings. To detect neurochemicals, a potential is applied to recording electrodes, typically carbon fiber microelectrodes, using a triangular waveform starting from a holding potential of -0.4 V and linearly increased to a switching potential of +1.45 V. The ramp direction is reversed and the potential is returned to the holding potential. (**B**) The FSCV switching potential can be independently varied to simultaneously detect different neurochemicals at each recording channel. (**C**) The holding potential can be independently varied to detect different neurochemicals at each recording channel. (**D**) The FSCV waveform applied to each channel can be optimized to detect different neurochemicals. A triangular waveform is optimized for detection of dopamine. An N-shaped waveform is optimized for detection of serotonin.

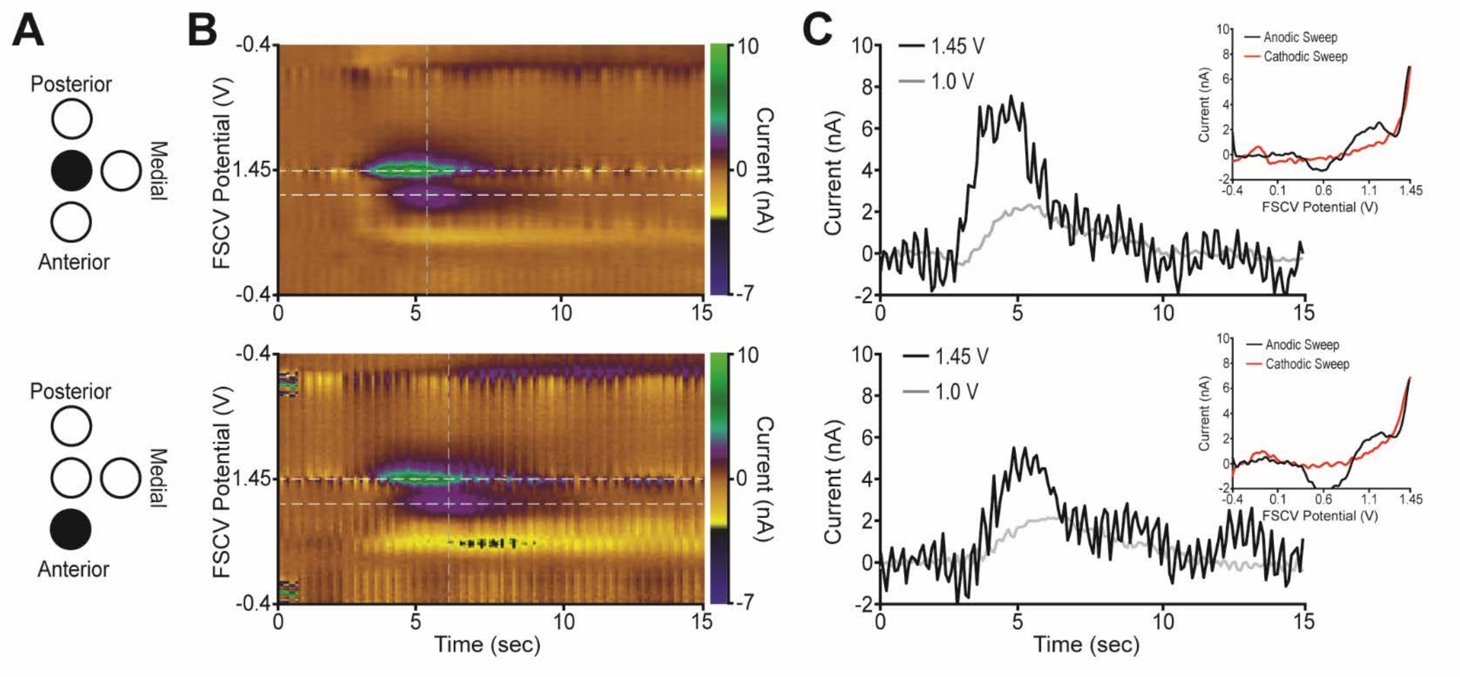

Figure S3. Adenosine release recorded at two different recording locations in the pig dorsal striatum. Data is recorded in the same animal and recording locations as presented in Figure 3. **(A)** Recording locations for each electrode which correspond to the electrode positions specified in Figure 3B. **(B)** Pseudocolor plots showing FSCV data for two potential spontaneous adenosine releases. **(C)** The time-series response for the same data shown in (A) of the two adenosine oxidation peaks at +1.45 V and +1.0 V, respectively. The background subtracted cyclic voltammograms recorded at the peak of the adenosine oxidation response (vertical dashed lines in the pseudocolor plots) are shown in the inset panels.

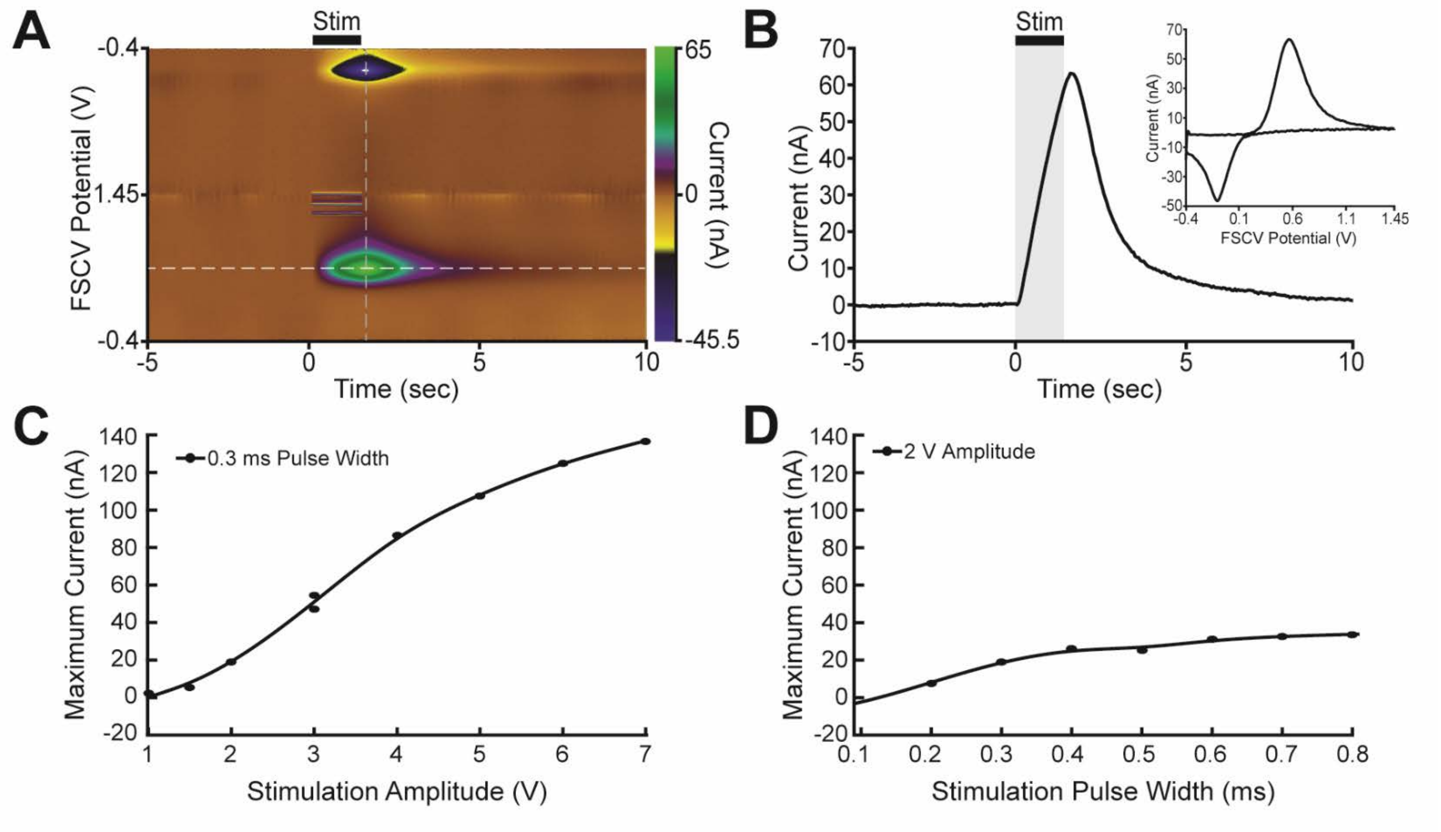

Figure S4. Dopamine release recorded in the dorsal striatum of a non-human primate (NHP) model of substantia nigra pars compacta (SNc)/ventral tegmental area (VTA) stimulation. (A) A pseudocolor plot showing FSCV data for one example of a stimulation-evoked dopamine release recorded with *WINCS Harmoni* in the NHP dorsal striatum with two seconds of 130 Hz, 0.3 ms pulse width, 3 V biphasic stimulation. **(B)** The time-series response of the dopamine oxidation current for the same data shown in (A) as well as the background subtracted cyclic voltammogram recorded at the peak (vertical dashed line in the pseudocolor plot) of the dopamine response (inset). **(C)** The relationship between stimulation amplitude and the observed maximum dopamine oxidation current response. **(D)** The relationship between stimulation pulse width and the observed maximum dopamine oxidation current response. All responses shown in (C) and (D) were observed with two seconds of stimulation at 130 Hz and stimulation parameters were presented in a random order with three minutes between stimulations.

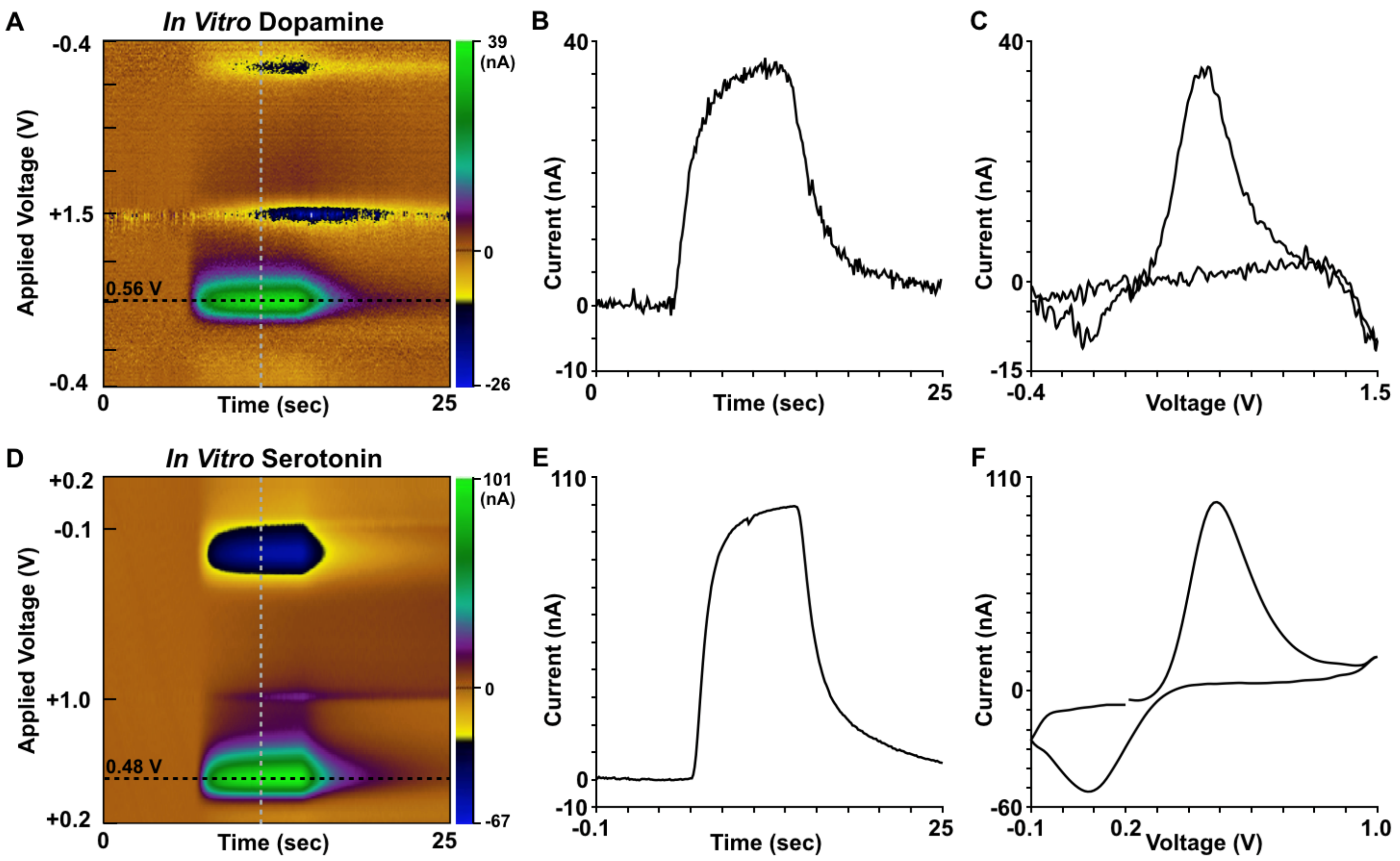

Figure S5. *In vitro* measurements of dopamine and serotonin. (A) A pseudocolor plot showing FSCV data for one example of a 1 µM bolus injection of dopamine recorded with *WINCS Harmoni* during flow injection analysis. **(B)** Time-series response of the dopamine oxidation current for the data shown in (A). **(C)** Background subtracted cyclic voltammogram recorded at the peak (vertical dashed line in the pseudocolor plot) of the dopamine response. **(D)** A pseudocolor plot showing FSCV data for one example of a 0.5 µM bolus injection of serotonin recorded with *WINCS Harmoni* during flow injection analysis. **(E)** Time-series response of the serotonin oxidation current for the data shown in (D). **(F)** Background subtracted cyclic voltammogram recorded at the peak (vertical dashed line in the pseudocolor plot) of the serotonin response. The current scale for the serotonin measurement is more than twice as large as the one presented for the dopamine signal, accounting for the seemingly different levels of noise in the signals.

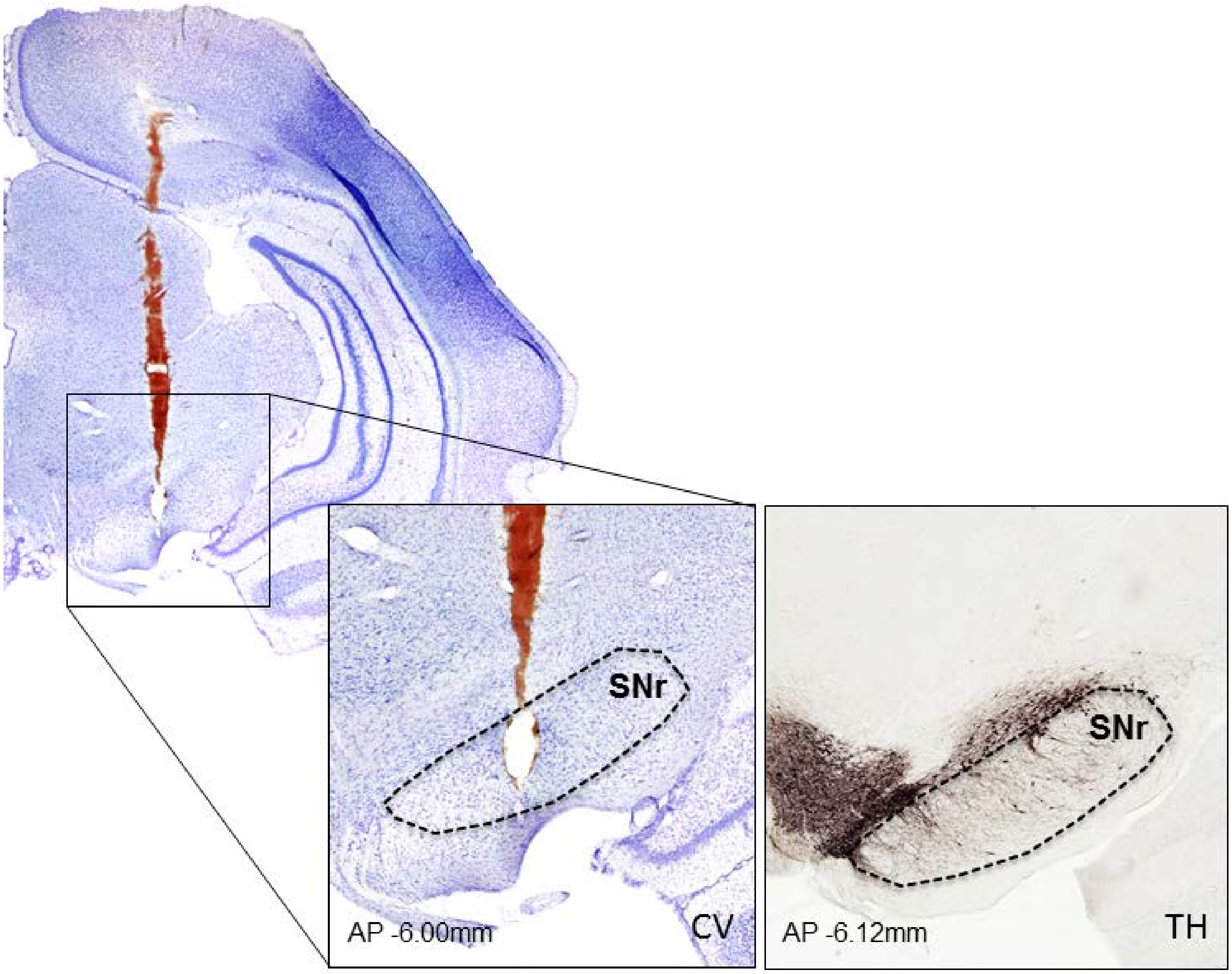

Figure S6. Histological verification of the serotonergic target within the substantia nigra pars reticulata. Cresyl violet stain was performed on the rodent brain to depict the track generated by the carbon fiber microelectrode (CFM) as it approaches the substantia nigra pars reticulata (SNr). A current was delivered through the recording electrode, leaving behind a marked lesion (<40μm) to indicate the exact placement of the electrode tip. Immunohistochemistry of tyrosine hydroxylase (TH) was also performed to provide additional confirmation of the anatomical structure of SNr in a brain slice 0.12mm posterior to the site of recording. The presence of stimulation-evoked serotonin was verified using the selective serotonin reuptake inhibitor fluoxetine.

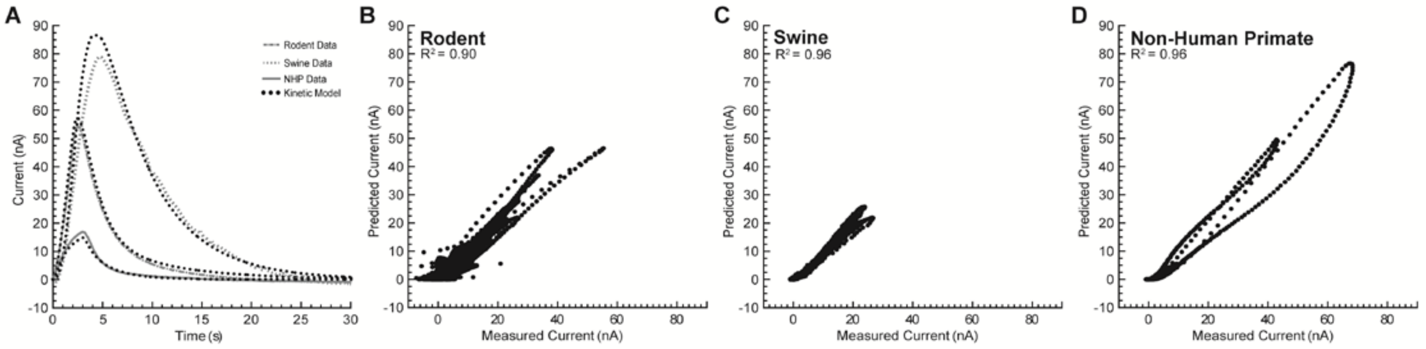

Figure S7. Predictive ability of the artificial neural network model. (**A**) Typical measured and predicted dopamine oxidation currents recorded in the striatum in response to medial forebrain bundle stimulation (rodent) or substantial nigra pars compacta/ventral tegmental area stimulation (swine and non-human primate [NHP]). (**B, C, D**) Regression analysis between all measured and predicted dopamine oxidation currents from the test data set for rodent (n=12), swine (n=4), and non-human primate (n=1) subjects, respectively.

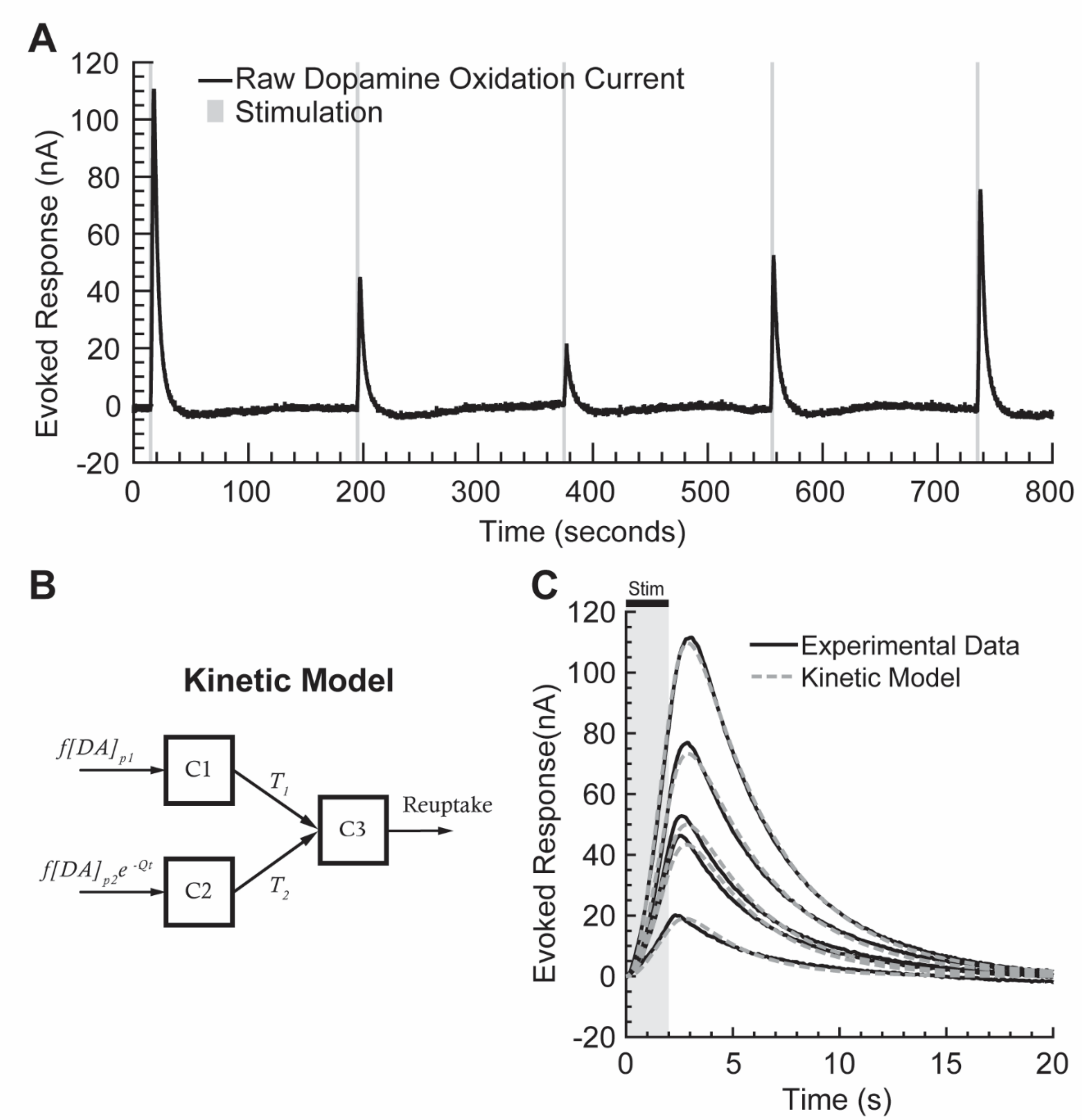

Figure S8. Mathematical characterization of medial forebrain bundle (MFB) stimulation-evoked striatal dopamine (DA) release. (**A**) Example of DA oxidation current recorded in the rodent striatum during a randomized series of electrical stimulations applied to the MFB. (**B**) Compartment model used to describe the kinetics of DA release; C1, C2, and C3 are compartments 1, 2, and 3 respectively; f is the frequency of stimulation; $[DA]_{p1}$ and $[DA]_{p2}$ are DA concentration increases that occur in C1 and C2 with each pulse of stimulation; R is the rate of attenuation of release into C2 with time; $T_1$and T_2 are transfer rates between compartments. (**C**) Fit of the DA model kinetics to the stimulation evoked-responses shown in panel (**A**).

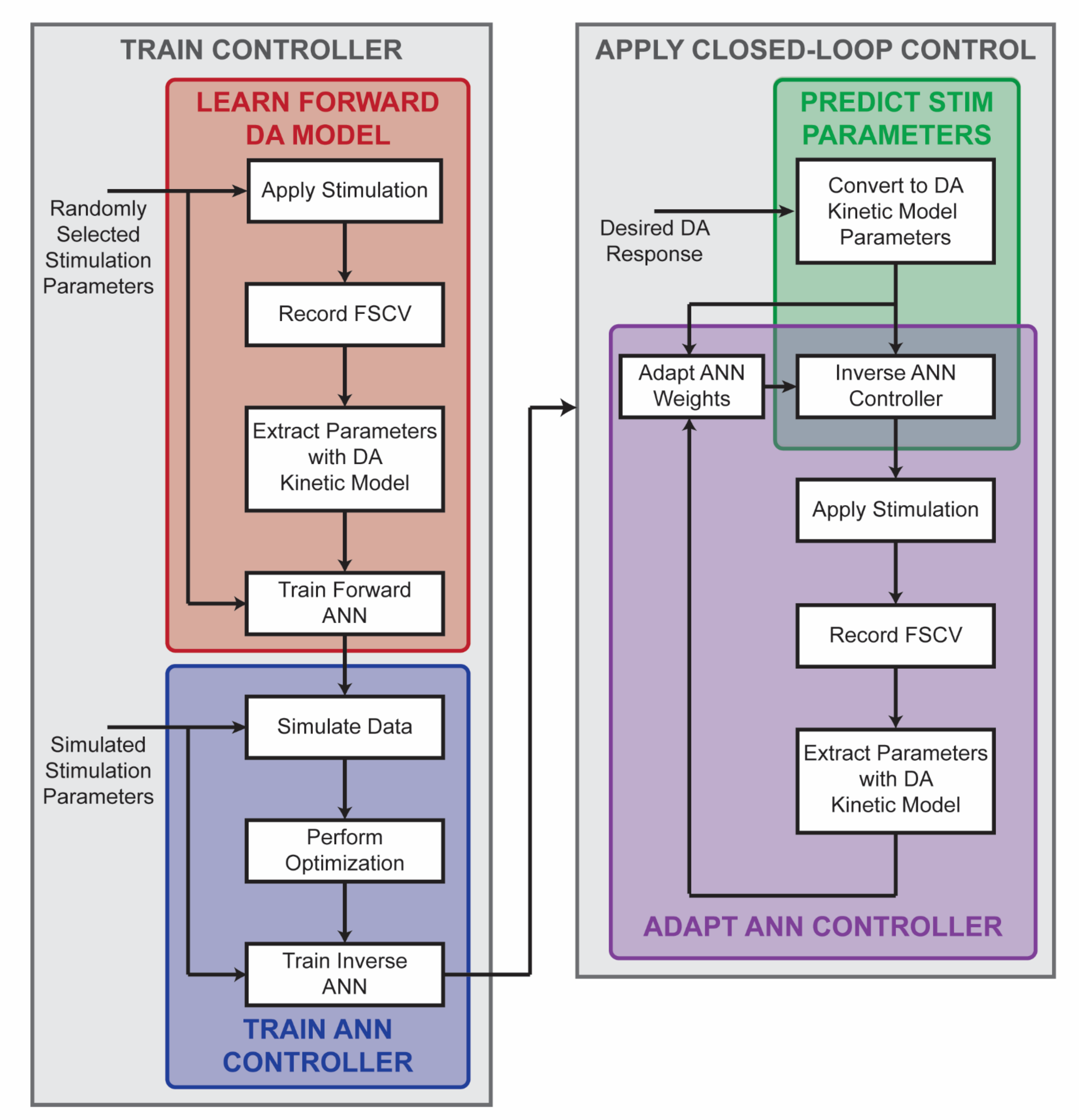

Figure S9. Predictive controller for stimulation-evoked neurochemical release. A flow diagram depicting the training of the artificial neural network (ANN) controller is shown on the left. ANN prediction of stimulation parameters and adaptation of the ANN controller in response to errors between the target and evoked dopamine (DA) response are shown on the right.

Table S1. Stereotactic coordinates for fast scan cyclic voltammetry (FSCV) and deep brain stimulation (DBS) electrodes in the rodent model. DBS electrodes were implanted in the medial forebrain bundle (MFB). Neurochemical recording FSCV electrodes were implanted in the striatum and substantia nigra pars reticulata (SNr). Coordinates are given in mm from bregma (0, 0, 0 in Paxinos and Watson, 1998) in the anteroposterior (AP), mediolateral (ML), and dorsoventral (DV) directions.

	**AP (mm)**	**ML (mm)**	**DV (mm)**
**DBS (MFB) for striatal recordings**	-4.6	+1.2	-8.7
**DBS (MFB) for SNr recordings**	-2.5	+1.7	-8.0
**FSCV (Striatum)**	+1.2	+2.0	-5.5
**FSCV (SNr)**	-4.8	+2.0	-8.0

Table S2. Range of biphasic stimulation parameters used to evoke dopamine release responses in the rodent, swine, and non-human primate (NHP) models of deep brain stimulation.

	**Frequency (Hz)**	**Pulse width (μs)**	**Amplitude**	**Duration (s)**
**Rodent**	60-100	0.1-2.0	0.01-0.80 mA	1-2
**Swine**	130	0.1-1.1	1.0-8.0 V	2-3
**NHP**	130	0.1-0.8	1.0-7.0 V	3
